# Solvent Free Fabrication of Micro and Nanostructured Drug Coatings by Thermal Evaporation for Controlled Release and Increased Effects

**DOI:** 10.1371/journal.pone.0040746

**Published:** 2012-08-01

**Authors:** Eman S. Zarie, Viktor Kaidas, Dawit Gedamu, Yogendra K. Mishra, Rainer Adelung, Franz H. Furkert, Regina Scherließ, Hartwig Steckel, Birte Groessner-Schreiber

**Affiliations:** 1 Functional Nanomaterials, Institute for Materials Science, Christian Albrechts University Kiel, Kaiserstrasse, Germany; 2 Department of Pharmaceutics and Biopharmaceutics, Christian Albrechts University Kiel, Grasweg, Kiel, Germany; 3 Department of Operative Dentistry and Periodontology, Christian Albrechts University Kiel, Arnold-Heller- Kiel, Germany; RMIT University, Australia

## Abstract

Nanostructuring of drug delivery systems offers many promising applications like precise control of dissolution and release kinetics, enhanced activities, flexibility in terms of surface coatings, integration into implants, designing the appropriate scaffolds or even integrating into microelectronic chips etc. for different desired applications. In general such kind of structuring is difficult due to unintentional mixing of chemical solvents used during drug formulations. We demonstrate here the successful solvent-free fabrication of micro-nanostructured pharmaceutical molecules by simple thermal evaporation (TE). The evaporation of drug molecules and their emission to a specific surface under vacuum led to controlled assembling of the molecules from vapour phase to solid phase. The most important aspects of thermal evaporation technique are: solvent-free, precise control of size, possibility of fabricating multilayer/hybrid, and free choice of substrates. This could be shown for twenty eight pharmaceutical substances of different chemical structures which were evaporated on surfaces of titanium and glass discs. Structural investigations of different TE fabricated drugs were performed by atomic force microscopy, scanning electron microscopy and Raman spectroscopy which revealed that these drug substances preserve their structurality after evaporation. Titanium discs coated with antimicrobial substances by thermal evaporation were subjected to tests for antibacterial or antifungal activities, respectively. A significant increase in their antimicrobial activity was observed in zones of inhibition tests compared to controls of the diluted substances on the discs made of paper for filtration. With thermal evaporation, we have successfully synthesized solvent-free nanostructured drug delivery systems in form of multilayer structures and in hybrid drug complexes respectively. Analyses of these substances consolidated that thermal evaporation opens up the possibility to convert dissoluble drug substances into the active forms by their transfer onto a specific surface without the need of their prior dissolution.

## Introduction

Important tasks for pharmaceutical engineering are the molecular scale mixing and structuring of pharmaceutical drugs for increased solubility. This includes coating of implants for multifunctional applications, or creation of hybrids/multilayers from different drugs and carriers for designing the controlled and sequential release. However, due to the nature of fluids this is difficult to achieve with conventional solvent based methods. Furthermore, the vast majority of newly discovered drugs result in molecules of poor solubility and currently only less than 10% of new drug substances have both high solubility and permeability [1], [2]. In general, therapeutic effectiveness of a drug depends on the bioavailability and hence on the solubility of drug molecules. Solubility is one of the most important parameters to achieve the desired concentrations of drugs in systemic circulation for a pharmacological response. Moreover oral absorption of the so-called biopharmaceutical classification system class II drugs is solubility-limited and is estimated to account for about 30% of both commercial and developmental drugs [3], [4]. The chemistry of a drug by itself is not alone responsible for its effect, it is mainly controlled by its physical structure [5]. The microstructure of the active components like the degree of crystallinity, combination with excipients like in co-crystals [6] or the covering with protective layers like poly-lactic-co-glycolic acid (PLGA) in order to prevent untimely disintegration are examples of internal or lateral structuring that are nowadays utilized to modify the mode of action of a drug.

From an engineering point of view the control over the drug particle size is very essential, as a smaller particle size (particularly in nanodimensions) leads to higher rates of dissolution [7], [8] due to large surface to volume ratio. Therefore appropriate and versatile fabrication method is required to formulate drugs in desired manner. Conventional drug micronization techniques, such as milling, grinding, and spray drying [9] are unable to provide a high level of control and often use toxic solvents, high temperatures, or mechanical stress possibly causing degradation of the drugs. Furthermore, residues of toxic solvents could remain in the products after processing, making them unsuitable for pharmaceutical applications [10], [11]. Equally, methods used for the encapsulation of drugs within polymer particles, such as phase separation, spray drying, and double emulsion techniques, are beset with the same problems [10]. Ideally, a drug should exert its pharmacological activity only at the target site, using the lowest possible concentration and without negative effects on non-target compartments [12] which can be effectively achieved by a proper drug structuring in terms of crystallinity and the layered structures. For example using nanostructured chemotherapeutic agents-this so-called enhanced permeability and retention (EPR) effect [13]- is dependent on their reduced size with that being enriched in the tumour by passive targeting. Another application is in drug eluting stents, where a precise control of the release kinetics can avoid late stent thrombosis [14]. But whenever solvents are involved, formulation of complex structured systems with two or more drugs, with a micro pattern or an embedding of a drug into metal/ceramic scaffold is very challenging task. Unintentional dissolution or mixing with already deposited material and surface tension effects like the so called “coffee stain effect” [15] (the ring-like agglomeration of precipitate due to drying droplets) prevent the proper and controlled structuring of drugs. Furthermore, the conventional coating of implants with drugs by spray coating or dipping to equip them with specific antibacterial substances [16], [17] often results in poor adhesion, depending on the wettability of the implant surface with the drug solution and the drying process.

In this work, we introduce a versatile approach for formulating micro-nanostructured solvent free drug delivery systems in various forms by using conventional physical vapour deposition technique. Thermal evaporation (TE) processes [18] have already been used to deposit individual atoms or molecules to form micro or nanostructured thin films since the last 50 years and to the best of our knowledge it has not yet been published in any scientific paper for the deposition of pharmaceutical drugs. The main advantage of thermal evaporation process is that it is a solvent free technique and can be routinely used to fabricate specimens which require a shape control down to the nanoscale. As a bottom up growth technique, atom by atom or molecule by molecule (depending upon the material) can be evaporated and assembled on the desired substrates to larger units and in new structures. The creation of multilayers or a patterning of the drug deposit are easily possible with the help of lithographic techniques or employing shadow masks. Even without further structuring, the internal structure of the deposited drug substance can be controlled [19]. Only by controlling the deposition parameters like evaporation rate [20], oven or substrate temperature, one can tune the degree of organization of the drug deposit from amorphous to single crystalline and the surface roughness from atomically flat to macroscopically rough. For higher dissolution rates and thus higher efficiencies in their e.g., antimicrobial of antifungal effects, the pharmaceuticals can be tuned towards an amorphous structure which is easy to dissolve. Thin film growth using physical vapor deposition in vacuum is well understood for decades [21] in which. the evaporated atoms or molecules impinge on the surface and immediately transfer their energy to the surface, followed by a substrate temperature driven diffusion. Nucleation and island formation occurs if evaporated atoms or molecules meet on the surface, starting crystal growth on the surface. Substrate temperature and deposition rate are the controlling parameters to tune the structure of the deposit from amorphous to crystalline. While thermal evaporation is mainly used for inorganic or ceramic materials [22], a widespread variant of the thermal evaporation in vacuum known as thermal organic vapor phase deposition, is commercially used for the fabrication of organic light emitting diodes (OLED) [23] for flat panel displays. Even though organic molecules for light emitting displays are of similar complexity like pharmaceutical molecules, surprisingly, their direct fabrication by thermal evaporation is not yet used in pharmaceutical engineering and also the growth mechanism is not well understood. The methods applied for the deposition of pharmaceutical molecules are manifold, but in all the cases they use solvents e.g., in spray coating [24], in supercritical point drying [25], [26], or in matrix assisted pulsed laser deposition (PLD) [27]. Impressively, PLD was already used to deposit complex proteins like glucose oxidase but again only if they were dispersed in a liquid as solvent prior to deposition. Therefore to overcome with these problems, we demonstrate here the versatile fabrication of solvent free micro-nanostructured pharmaceutical drugs in a controlled manner on various substrates. The morphological and structural investigations of fabricated micro-nanostructured pharmaceutical drugs are presented and the growth kinetics is also discussed. Preliminary results for multifunctional applications of these fabricated solvent-free pharmaceutical drugs are also presented.

## Results and Discussion

**Figure 1:**
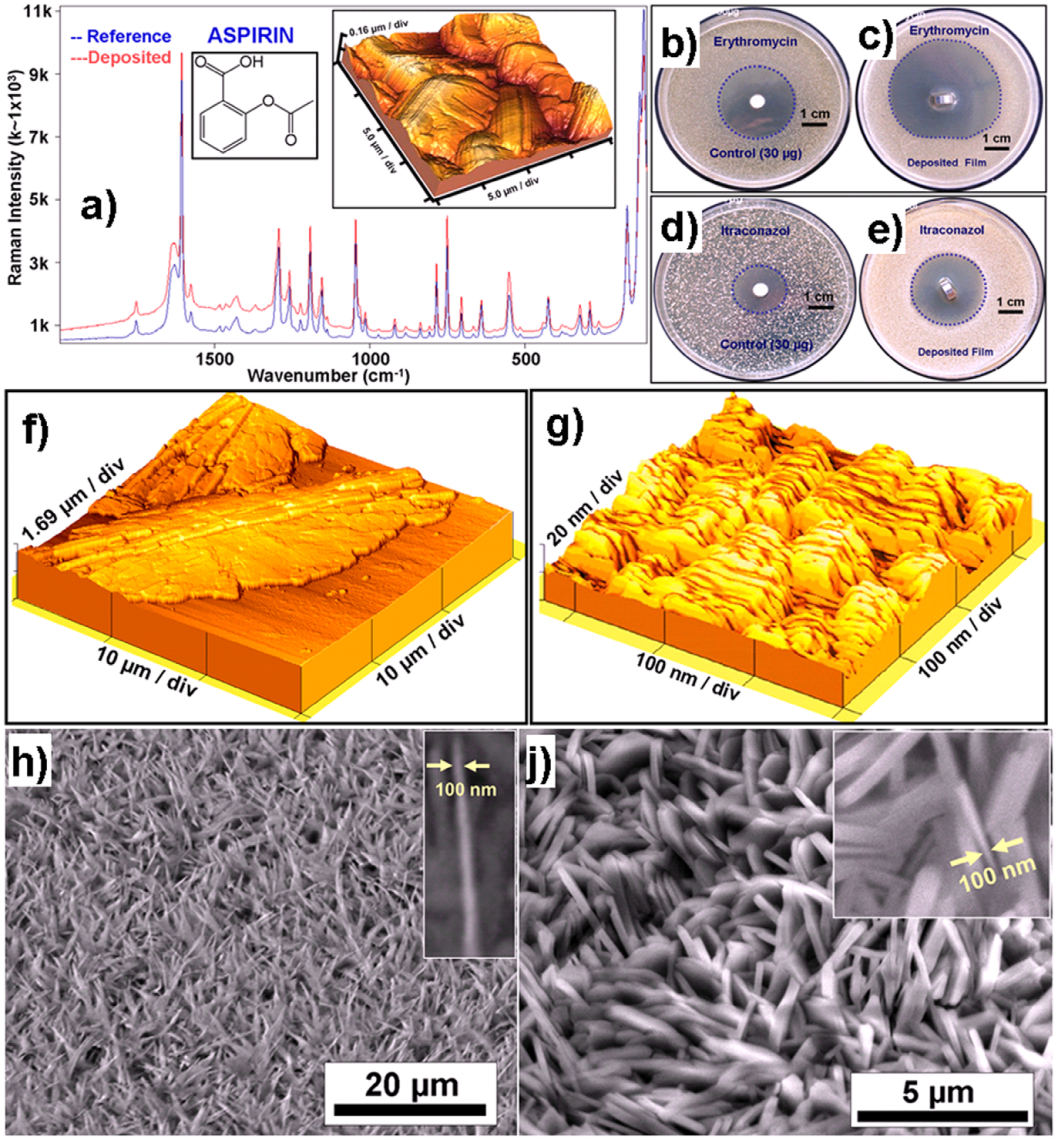
Analysis of TE deposited pharmaceutical films on Ti-substrates. a) Raman spectra of the deposited and reference acetyl salicylic acid (ASS), showing clear Raman peaks in the TE deposited ASS. 3D AFM image in the figure a, from the TE ASS sample, shows the micro-crystallites of ASS. b–e: The antimicrobial activity of Erythromycin (b: control, c: TE deposited) against Staphylococcus aureus and of Itraconazole (d: control, e: TE deposited) against Candida albicans demonstrated in agar diffusion tests. The activity is significantly increased in the case of specimen deposited by TE. f) Tuneable crystallinity: 3D Atomic force microscope image of Nipasol grown on Ti substrate at room temperature resulting in a much larger crystal size as compared to that deposited at −100°C as shown in g) which is nano-crystalline. h) & j) SEM images of nanostructured Cholesterol h) and Tetracaine-HCl j) drugs respectively fabricated on different substrates by thermal evaporation showing nanoscale spikes, see insets. The nanospike or platelet like geometries result from the growth kinetics.

In order to evaluate the suitability of thermal evaporation for pharmaceuticals, we tried the approach on several (>30) pharmaceutical substances (table 1) under different parameters and as a proof of principle several application examples have been demonstrated here. The evaporation temperatures were always chosen between melting and boiling temperature of the drugs and we have observed that the vast majority of drugs (>80%) showed (table 1) the deposition of high quality crystalline films without decomposition. The formation of micro-nanoscale pharmaceutical crystals on the substrate by thermal evaporation already indicates a high purity which was further confirmed in several cases by Raman spectroscopy investigations. For example the Raman spectra of corresponding to bulk and thermally evaporated Aspirin are shown in figure 1a which clearly demonstrate that after thermal evaporation the micro-nanostructured film exhibits almost same chemical structure as compared to it’s bulk. The 3D atomic force microscopy (AFM) image of the deposited Aspirin film in the inset of figure 1a depicts the formation of large Aspirin crystals. Similar investigations for several drug materials (table 1) were performed and most of them were found to be structurally intact (table 1). In the case of antimicrobial substances, the nanostructured thin films were directly deposited on titanium substrates and their antibacterial or antifungal activities were tested. Table 1 shows the complete list of different drug materials tested for micro-nanostructuring in thermal evaporation process and the corresponding remarks about the structural evolutions as well as extent of inhibition areas. It has been observed that even very poorly water soluble substances such as Metronidazole, Erythromycin and Itraconazole exhibit pronounced activities when deposited by thermal evaporation. Some of the agar test plates with thermally evaporated antimicrobial agents (Erythromycin & Itraconazole) in comparison with their control samples can be seen in figures 1b,c,d,e, where they are compared with their higher doses (30 µg) as conventional control. The antimicrobial activities of Erythromycin control sample and its thermally evaporated thin film against Staphylococcus aureus (ATCC 6538) are shown in fig. 1(b) and 1(c) respectively and a clear increase in area of inhibition zone for thermally deposited Erythromycin thin film can observed. In similar manner the antifungal activities of Itraconazole control sample and its thin film against Candida albicans (ATCC 10231) are demonstrated in fig. 1(d) and 1(e) respectively where one can also observe an increase in the area of inhibition zone for the case of TE thin film. This significant increase in the area of inhibition zones for thermally evaporated Erythromycin (fig. 1c) and Itraconazole (fig. 1e) with lower doses (<30 µg) in comparison with the conventional control sample (30 µg) can be explained by an increased dissolution which is due to large surface area and an almost amorphous assembly of the deposited drugs.

**Table 1:** Overview of thermally evaporated different drug substances.

Test Material; *Indication and potential use of solvent free nanostructuring by TE*	Melting point at atmosphere (typical evaporation temperature range) [°C]}	Deposition remarks	Maximum extent of inhibition area (cm^2^) [TE deposited on titan plates]	Maximum extent of inhibition area (cm^2^)[Control (paper, 30 µg/disc)]
**1. Chloramphenicol;** *Antibiotic,* *Increased solubility*	150 (∼90–118)	Crystalline deposits	2.55 (S.* aureus); Amount<30 µg	1.36
**2. Erythromycin (base);** *Antibiotic, Increased oral* *bioavailability*	190–193 (∼100–137)	Amorphous and crystalline deposits; Raman spectra	3.11 (S. aureus); Amount<30 µg	1.38
**3. Metronidazole;** *Antibiotic, Dental implant coating*	159–163 (∼100–194)	Crystalline deposits; Raman spectra	2.06 (S. aureus); Prove of prin-ciple, amount not further quantified	1.67
**4. Propyl-p-hydroxybenzoate (Nipasol);** *Preservative, pre-preserved container*	95–98 (∼85–165)	Crystalline deposits	1.16 (S. aureus); Prove of prin-ciple, amount not further quantified	Under progress
**5. Neomycin sulphate;** *Antibiotic, Surface coating of* *wound dressings/implants*	250–260 (∼160–300)	Amorphous and crystalline deposits	0.57 (S. aureus) Amount<30 µg	0.68
**6. Novobiocin sodium salt;** *Antibiotic, Surface coating of* *wound dressings/implants*	215–220 (∼150–250)	Amorphous and crystalline deposits	3.95 (S. aureus) Amount<30 µg	1.64
**7. Tetracycline hydrate;** *Antibiotic, Device coating*	170–175 (∼100–223)	Crystalline deposits	1.44 (S. aureus) Amount∼3.25 µg	1.82
**8. Tetracycline HCl;** *Antibiotic, Device coating*	220–223 (∼119–255)	Crystalline deposits	1.65 (S. aureus) Amount<30 µg	Under progress
**9. Vancomycin HCl;** *Antibiotic, Increased oral bioavailability*	185 (∼100–197)	Amorphous and crystalline deposits	0.67 (S. aureus) Amount<30 µg	0.80
**10. Clotrimazole;** *Antimycotic, Improved nail coating*	147–149 (∼89–164)	Crystalline deposits; Raman spectra	1.76 (Candida albicans); Proveof principle, amount notfurther quantified	1.72
**11. Itraconazole;** *Antimycotic, Increased oral bioavailability*	166,2 (∼100–232)	Amorphous and crystalline deposits	1.82 (Candida albicans) Proveof principle, amount notfurther quantified	0.97
**12. Ketoconazole;** *Antimycotic, increased solubility*	146 (∼88–226)	Amorphous and crystalline deposits	2.83 (Candida albicans)Amount <30 µg	1.07
**13. Pindolol;** *Beta blocker,* *Contact lens coating*	167–171 (∼110–215)	Crystalline deposits/Raman Spectra		
**14. Pilocarpine HCl;** *Parasympatho-mimetic, Contact* *lens coating*	200–203 (∼117–250)	Crystalline deposits/Raman Spectra		
**15. Cholesterol;** *Excipient, Biocompatible protection layer*	148–150 (∼90–165)	Crystalline deposits		
**16. PLGA** *Excipient, Controlled release layer*	224–226 (∼ 150–300)	Amorphous deposits		
**17. 5-Fluorouracil;** *Antimetabolite (Anticancer)*	283 (∼115–250)	Crystalline deposits		
**18. Sulfathiazole;** *Oral and* *topical antibiotic*	200–203 (∼110–250)	Crystalline deposits		
**19. Acetyl salicylic acid (ASS);** *Non* *steroidal anti-inflammatory* *drug*	135 (∼90–140)	Crystalline deposits		
**20. Paracetamol;** *Non steroidal anti-inflammatory drug*	168 (∼97–130)	Crystalline deposits		
**21. Diclofenac sodium;** *Non steroidal anti-inflammatory drug*	284 (∼135–270)	Crystalline deposits/Raman spectra		
**22. Ascorbic acid** *Antioxidant*	190–192 (∼123–200)	Crystalline deposits		
**22. Tetracaine HCl;** *Local anaesthetic*	149 (∼114–220)	Crystalline deposits		
**23. Trimethoprim;** *Antibiotic*	199–203 (∼100–170)	Amorphous and crystalline deposits		
**24. Indometacin;** *Non steroidal anti-inflammatory drug*	155 (∼150–190)	Amorphous and crystalline deposits		
**25. Polyglycolic acid;** *Biodegradable polymer, Control drug release*	225–230 (∼160–200)	Amorphous and crystalline deposits		
**26. Adipic acid;** *Excipient*	152.1(∼119)	Crystalline deposits		
**27. Caffeine;** *Psychoactive stimulant drug, Excipient*	227–228 (∼120–250)	Crystalline deposits		
**28. Lactose Excipient**	223	Deposition not possible		
**29. Polyethylene glycol;** *Excipient*	49–53	Deposition not possible (decomposed to gas)		
**30. Chlorhexidine;** *Antiseptic*	134–136	Decomposes, droplets on the surface of substrate		
**31. Triclosan;** *Antibacterial and antifungal agent*	56–58	Deposition not possible (Degrades to dioxin)		

First part (1–12) lists the materials which has been successfully nanostructured by thermal evaporation and have been tested with disk diffusion method. The second part (13–27) lists the successfully deposited materials however the biological tests are under progress. The last part (28–31) lists the pharmaceutical substances which are not suitable for thermal evaporation as they are decomposed during deposition. (*Staphylococcus).

In view of the variety of molecules being capable of thermally evaporated found here, there seems to be no regularity which of the complex molecules stay intact and which decompose. A general precondition for TE process is that the thermal energy (here >300°C meaning ∼0.05 eV) injected into the molecules, stays far away from the range of the intermolecular binding energies of typically 2–7 eV, which seems to be fulfilled. For a better comparison of the process, it should be understood in which energy steps a single molecule can leave the different bonding force fields in its surrounding liquid and enter the vapor phase, or otherwise which energy barriers have to be taken in sequence to decompose a molecule. Such sophisticated calculations are difficult to carry out and not yet known, the large variety of possibilities for the molecules to wiggle, swing or rotate in its liquid state complicates it to foresee which of the molecules find their way into the gas phase and which decompose. Even if the decomposition occurs in some fragments a recomposition on the substrate could occur. Our arbitrary choice of molecules seems to confirm this, but an obvious prediction of the suitability for TE needs significant efforts to be done in future. It must be noted that relatively large molecules like Pindolol, Erythromycin or Tetracaine-HCl also remain intact as confirmed by Raman investigations. In principle, the evaporation of the molecules may have a positive side effect by purifying the evaporated substance, as the contaminants with a lower melting point are evaporated early when the substrate is still covered by a shutter of the evaporation oven. Other way round those contaminants higher melting point will not evaporate at all.

### Control Over the Crystallinity of the Deposited Drug Films

With the molecules being suitable for evaporation, the full versatility of thermal evaporation can be utilized. This means that by changing the deposition rate, the crystallinity of the deposited film can be controlled in a wide range from amorphous to almost single crystalline. Figures 1f and 1g illustrate this with an example of a Nipasol drug film which is nano-crystalline in the case of deposition at −100°C to a micro-crystalline film deposited at room temperature. With a liquid N_2_ cooled substrate holder, even completely amorphous films can be deposited with a relatively high free surface energy. Moreover the thickness of the coating can be varied, for the here used pharmaceutical drugs from a few nm to many µm simply by changing the evaporation rate and deposition time as confirmed by AFM and profilometer measurements. Fabrication of micro-nanostructured coatings of pharmaceutical drugs on desired substrates in a controlled manner is a very promising aspect of thermal evaporation technique. For example the deposition of nanostructures of Cholesterol and Tetracaine-HCl drugs in the range of ∼100 nm to ∼500 nm thicknesses are shown in figures 1h) and 1j) respectively. These structures typically exhibit high surfaces which enable them to dissolve faster as compared to their bulk counterparts and also they are solvent free. As it is typical for TE, the surface of the substrate has strong influence on the structure in terms of crystallinity and shape of the deposited drug.

**Figure 2:**
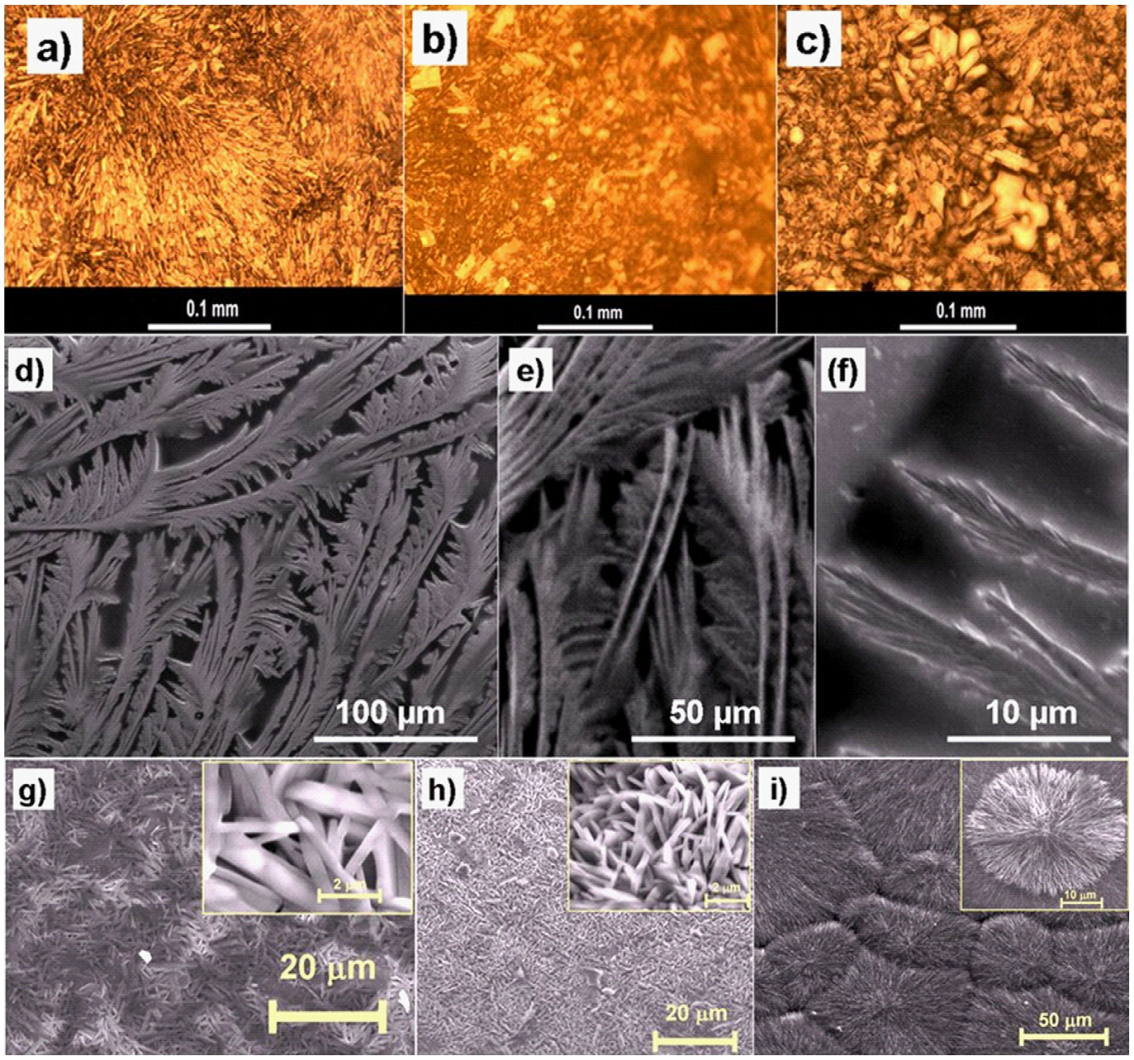
Representative optical microscopy and SEM images of various active pharmaceutical ingredients grown on different substrates synthesised by TE process (a–c) optical images of various Pilocarpine-HCl micro- or nanostrucured morphologies on different substrates like glass (a), polymer foil (b), Ti (c), d–f: SEM images of Ascorbic acid deposited on Si substrate at lower and higher magnifications; g–i: SEM images of Tetracaine-HCl nanostructures deposited on titanium substrate (g), silicon wafer coated with 20 nm Au thin film (h) and silicon coated with 4 nm Au thin film (i). The inset images inside figures 2 g) to 2 h) show their magnified SEM view of deposited drug respectively.

Fig. 2a, 2b, 2c show the morphological evolutions of TE deposited Pilocarpine-HCl as active pharmaceutical drug on glass, polymer foil and titanium substrates respectively. It can be observed that substrate surface has strong influence on structural evolutions of the pharmaceutical drugs during thermal evaporation. Apart from the standard growth of nanoscale thin films with a certain degree of crystallinity, formation of rather complicated hierarchical structures may be triggered, as illustrated by SEM images for Ascorbic acid in fig. 2d, 2e, 2f at different magnifications. It must be noted that the hierarchical assembly of the drug structures down to the nanoscale leads to a huge surface to volume ratio which significantly increases the solution kinetic processes by giving more possibilities for drug molecules to step into the liquid phase and thus increase in solubility. Figures 2g, 2h, 2i show further examples for different nanoscale roughnesses and large surface areas formed by self organization during growth and appearance of thermally evaporated thin films of Tetracaine-HCl on different substrates as visualized by scanning electron microscopy. The inset images in fig. 2g), 2h) and 2i) are the corresponding higher magnification SEM images showing a clear visibility of microstructural patterns of the deposited Tetracaine-HCl drugs. The choice of substrate may comprise of a wide range of materials and even highly soluble substances can be employed to carry pharmaceutical thin films without the risk of side effects like dissolution of the substrate. A limitation is given for pharmaceutical drugs which are instable or altered in vacuum.

### Control Over the Lateral Structuring of the Deposited Drug Films

**Figure 3:**
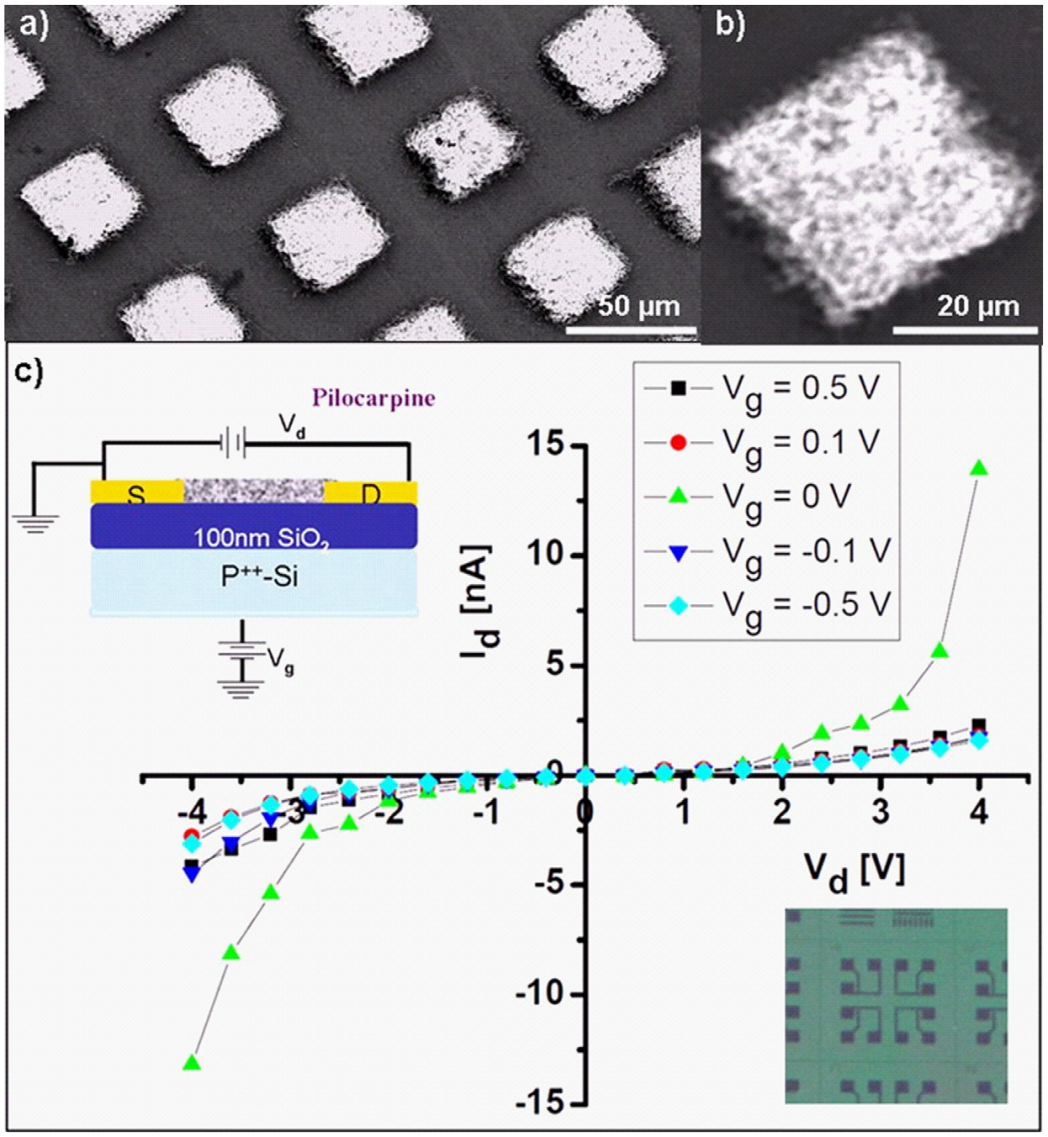
Lateral structuring of pharmaceutical substances. a) Representative SEM images of microstructured ASS morphology by deposition through a microscopic shadow mask. b) magnified SEM image of the square shaped of the deposited drug. c): current voltage response of a pharmaceutical field effect transistor (PFET) of Pilocarpine-HCl, cross section scheme (upper left) and part of the waver (lower right) used for the lateral structuring of the Pilocarpine-HCl PFET fabrication are shown as insets.

A lateral structuring i. e. the positioning of thermal evaporated thin drug films, is relatively straight forward by utilizing lithography techniques or shadow masks. Possible applications for laterally structured systems are smart or multifunctional pharmaceutical drug coatings for implants or contact lenses. Specific examples are contact lenses for drug delivery [28] which should not affect the vision and thus require a lateral structuring of the drug film. Selective area deposition of pharmaceutical drugs on contact lenses is possible with TE technique using a suitable masks. Such a structuring could also be used in complex implant coatings like stents or dental implants that require different drugs on the different zones in contact with bone, mucosa or the oral cavity. Bacterial adhesion is an important step in the pathogenesis of an infection (periimplantitis) sometimes leading to implant loss. Placement of different antibacterial active molecules on specific areas of the implant surface (depending on the intraoral contact to bone, mucosa or the oral cavity) will also be possible with thermal evaporation. To demonstrate these possibilities, fig. 3a and b show the SEM images at different magnifications of thermal evaporated Acetyl salicylic acid (ASS) through a microscopic shadow mask resulting in lateral structuring of the deposited drug. The feature of lateral structuring also gives the possibility to cover the different surfaces such as inner and outer walls of stents with different drugs. Another promising possibility could be to selectively coat the integrated silicon chips to design a pharmaceutical field effect transistor (PFET) for different applications ranging from new generations of organic electronic devices to biomedical engineering [29], [30]. Fig. 3c shows the basic layout and conductivity curves of a PFET based on Pilocarpine-HCl by deposition through a shadow mask directly on a silicon microchip. The top and bottom insets in fig. 3c demonstrate the schematic of PFET layout and the camera image of the microstructured Si chip used for fabrication of Pilocarpine-HCl PFET respectively. A gate voltage (V_g_) allows the blocking of a source (S)-drain (D) current. A clear change in the drain current (Id) can be seen at gate voltages slightly above 0 Volt which suggests that that it can be used as a switching electronic device. While other organic electronics like organic light emitting diodes are already established technology, a pharmaceutical field effect transistors (PFETs) does not exist so for to the best our knowledge. Here we just demonstrate the fabrication and functionality of PFET using Pilocarpine-HCl which in future could be of large interest for multifunctional applications in bioelectronics and biosensors. Visionary, these type of microstructured films could be used for multifunctional medical coatings allowing to read out the status of the drug layer like thickness. This 3D structuring may be used to build nano-or microscopic particles with a predefined shape which might be beneficial for special applications like particulate drug uptake [31]. In principle, the demonstrated techniques of microstructuring enable the formation of miniaturized drugs on micro- or nanoscopic carriers which can be utilized to overcome the blood brain barrier [32] or accumulate in cancer tumours due to the enhanced permeability and retention (EPR) effect.

### Sequential Deposition: Multilayers of Different Drugs

**Figure 4:**
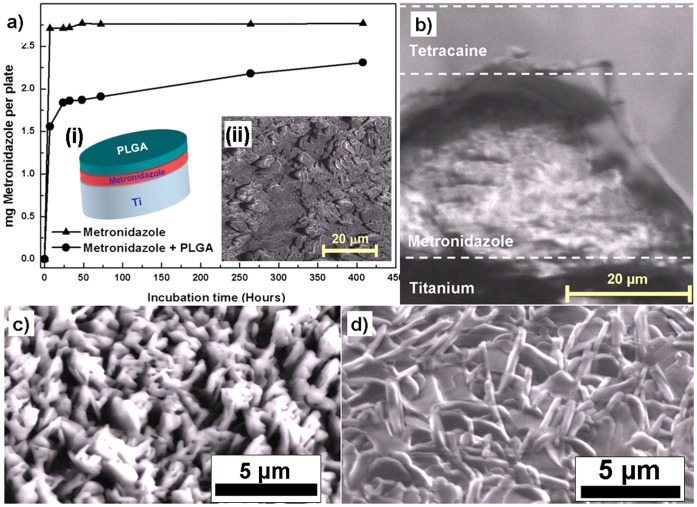
Multilayer and co-deposition experiments with pharmaceutical substances. a) Comparative dissolution profile of Metronidazole alone and Metronidazole covered with the PLGA thin layer. The insets (i) and (ii) in the fig. 4a show the schematic of the multilayer coating and SEM image of the TE deposited Metronidazole drug respectively. b) 3D SEM micrograph an a cross section cut in the structure of a double layer showing top layer of Tetracaine and second layer of Metronidazole deposited on Ti substrate, c) SEM Image of the nanocomposite of Tetracaine-HCl with Ag on the Si substrate fabricated by co-deposition from two sources simultaneously sources and d) SEM image of Tetracaine-HCl without silver for comparison.

Additionally, the appropriate fabrication of multilayers of pharmaceuticals controls the release kinetics of the drug which can find potential applications in drug deliveries etc. A fast dissolving drug can be protected by a second layer that needs to be dissolved first. Just for demonstration 2-layered structure using Metronidazole and PLGA on titanium substrate was fabricated by thermal evaporation technique and its release kinetics was studied with respect to incubation time which is shown in fig. 4a. It can be observed that the drug release occurs slowly if it is protected by another drug layer. The insets (i) and (ii) in fig. 4a show the schematic design for multilayer drug fabrication and SEM image of Metronidazole film respectively. In this case a thin film of Metronidazole, being rapidly dissolved after TE, is covered with PLGA by subsequent depositions from two sources on a polished Ti-substrate. From the release kinetics shown in Fig. 4a the effect of delayed drug release could be verified. Being a polymer, PLGA is expected to depolymerise during evaporation and to re-polymerise on the substrate. Additional to the delayed release, such multilayered systems may be used to release a combination of drugs in a desired time pattern, e.g., having a drug I for a few days, after complete dissolution of layer I, a subjacent drug II could take effect. This approach of fabricating multilayer pharmaceutical drugs by TE could be used for coronal stents, which first require an antiplatelet medication and subsequently an antiproliferative drug to prevent restenosis in coronary arteries and effect an optimal integration. The morphology at the interface between the individual layers also plays very important role in controlling the drug release kinetics and therefore the cross-section of Metronidazole-Tetracaine double-layer drug was investigated by SEM as visualized in fig. 4b. It is very clear from the cross-sectional view that individual layers maintain their structures during sequential thermal evaporation.

Another very promising route of a new drug design by thermal evaporation is the co-deposition of two different drugs from two separate evaporators simultaneously in a vacuum chamber. This allows the formation of a molecular scale mixing of pharmaceutical active substances or excipients to form nanostructured hybrid drugs which can exhibit synergetic responses at the same time. With conventional solution based processing, such a mixing requires very special preconditions, e.g., the possibility for co-crystallisation. Evaporation of molecules from two different sources typically gives no time for a demixing process on the substrate, as molecules directly loose most of their energy when they hit the substrate surface. Here, even amorphous mixtures can be formed with structurally incompatible drugs, leading to synergistic effects. The approach is very beneficial for controlling the dissolution of drugs and furthermore the solubility can also be significantly increased. If a molecule with poor ability to dissolve is only surrounded by molecules of good solubility, a total increase in dissolution can be effected. This is very beneficial in increasing the solubility of poorly soluble drugs by deposition of a low soluble molecules in parallel with a highly soluble one and with this forming a solid solution. The co-deposition approach also allows the creation of a molecular mixture in terms of a solid solution from nearly every thermally evaporated drug (overviewed table 1). To show an example for such a combination, the efficacies of the above mentioned antimicrobial agents such as Erythromycin and Itraconazole were evaluated in combination. In agar plate diffusion tests, both antifungal and antibiotic effect of these combined deposits could be confirmed and were in the same degree as for the individual substances (as already shown in Fig. 1 (b, c) and 1(d, e) for Erythromycin and Itraconazole respectively. ‘From our first biological tests as starting it is obvious that thermally evaporated substances can in principle exhibit higher activities as compared to their conventional control sample. However further biological experiments with other pharmaceuticals should be performed to reveal the full potential of thermal evaporation for pharmaceutical engineering.

A completely new way of pharmaceutical engineering is the combination of inorganic materials with pharmaceutical active substances. Such combinations could be potentially used as smart implant coatings that form nanoporous structures with medication filled pores in the inorganic material. In terms of the oral implant example given above, these molecules may be embedded in nanoscale sponge like ceramic structures by co-evaporation to have a potential depot function of antibacterial factors in order to reduce the risk for periimplantitis. An example for such co-evaporation is shown by SEM measurement in fig. 4c where Ag and Tetracaine-HCl are combined. For comparison, fig. 4d shows the SEM image of the deposited Tetracaine-HCl without silver. The inorganic part of the hybrid structures could be also used as active ingredient in wound dressings, e.g., silver nanoparticles are known for their antimicrobial effect [33].

In summary, thermal evaporation of pharmaceutical molecules can be utilized as a completely solvent free technique where drugs as powders or crystalline materials are directly formulated into desired nano or micro structures and shapes on preferred substrates. The performed deposition experiments and their structural analyses suggest that by thermal evaporation technique most of pharmaceutical substances (>80%) can be micro-nanostructured in different desired complex forms. The observed features of enhanced solubility and activity, controlled release kinetics of synthesized drugs seem to be a very powerful alternative for the traditional physical structuring of pharmaceutical drug molecules. The variety of new possibilities for pharmaceutical engineering utilizing thermal evaporation is expected to be very wide and will be very beneficial for many branches of medicine, pharmacy and engineering. Since a large number of drugs are accessible to the approach, thermal evaporation tests could result in reduction of dose and side effects for many approved drugs. The possibility of smart designing like hybrid, multilayer and/or implant coatings or solid solutions could lead to a re-assessment of insoluble molecules and thus to could be very promising for many new therapies. Here we have demonstrated that thermal evaporation technique can be versatily employed for micro-nanostructuring of different pharmaceutical drugs in various desired forms with increased responses. As an outlook, we would like to emphasize that thermal evaporation is not only limited to the drugs listed here but also can be employed for restructuring of unrestricted old pharmaceutical into new forms of medications with improved responses.

## Materials and Methods

### Selection of Drugs and Thermal Evaporation

Active pharmaceutical ingredients and excipients selected for this study were Nipasol, Tetracycline-HCl, Tetracycline hydrate, Chloramphenicol, Pilocarpine-HCl, Pindolol, Clotrimazole, Diclofenac Sodium, Erythromycin (base), Metronidazole, Itraconazole, Neomycin sulphate, Novobiocin Sodium, Vancomycin-HCl, Sulfathiazole, 5-Fluorouracil, Acetyl salicylic acid (ASS), Paracetamol, Trimethoprim, Indometacin, Tetracaine-HCl, Caffeine, Ascorbic acid and excipient materials and biodegradable polymers like Cholesterol, Adepic acid and Poly-lactic-co-glycolic acid (PLGA) and Polyglycolic acid (PGA) which are all commercially available and were purchased by common suppliers (Merck, Sigma Aldrich).

Thin films of active pharmaceutical materials were deposited by thermal evaporation in a vacuum chamber evacuated by a turbo molecular pump to 10^−5^ to 10^−7^ mbar on various substrates kept at room temperature and −100°C. The thermal evaporators are of Knudsen type equipped with metal or graphite crucibles. Deposition temperatures vary for different drugs and hence several drugs are deposited at several temperatures (see table 1 for details). For the standard procedure, the ovens were heated stepwise over several minutes towards the desired evaporation temperature. After having reached the deposition temperature, shutters were opened allowing the deposition for a chosen time, followed by closing the shutter and reducing the temperature. A pressure gauge as well as a thermocouple at the Knudsen cell and the sample holder were used to control the deposition process. For co-deposition, two ovens were mounted with an angle of 135° with respect to the sample holder that both ovens have an angle of 90° with respect to each other. The vacuum ovens are equipped with a mechanical shutter that allows a blocking of the evaporated molecular flux. For simultaneous organic/inorganic evaporation experiments, metals were evaporated at temperatures of ∼1000°C.

### Optical, Atomic Force, Scanning Electron and Raman Microscopies

Bright field optical micrograph images of deposited drugs on different substrates were taken by Zeiss optical microscope equipped with a Leica DFC 280 digital camera system at different magnifications. Atomic force microscopy (AFM) images were recorded with Thermo Microscope-Veeco autoprobe AFM equipped with an optical unit and electronic module in contact and non contact mode. Standard silicon nitride tips mounted on cantilevers were used. Philips-FEI XL30 SEM equipped with an EDAX EDX detector was used to analyse the structural geometry of nano- or micro- scale deposited drugs. In order to avoid the structural damage in the structures and the charging, acceleration voltages were kept as small as possible down to 2 kV and reduced currents were applied. In order to get the actual morphologies of thermal evaporated pharmaceuticals, no further coating (of carbon or gold thin film) was performed on deposited drugs. This was the reason that the SEM images with higher magnification appear less focused. Raman spectra of raw drugs as well as thermally evaporated layers on different substrates were recorded to ensure the identity of substance using a Senterra Raman Microscope (Bruker Optik GmbH, Bremen, Germany) with OPUS software (Bruker). For the raw material, the sample was prepared on a sample holder or on a mirror plate to obtain a flat sample surface for measurement. The sample was mounted under the objective and the sample surface was brought in focus using the light microscopic image. For all measurements, a 20x magnification is identified as suitable and in combination with a slit aperture of 50×1000 µm ensures collection of the highest possible Raman intensity. Collection of the Raman spectra has been performed in the dark field with 785 nm laser excitation (50 mW) at a resolution of 3–5 cm^−1^ resulting in a full spectrum from 80–3500 cm^−1^ with 10 seconds integration time and 2 co-additions. The Raman shift was calibrated automatically using the SURECAL option of the instrument. For analysis, the Raman band positions in the fingerprint region of the vacuum deposited drugs were compared with respect to the Raman spectrum of the raw material.

### Current-Voltage (FET) Response

Current-voltage (I-V) field effect transistor (FET) response of a nanostructured pharmaceutical drug fabricated directly on a microchip with gold contacts was measured by FET using a Keithley 6485 pico-amperemeter (optimised for measuring small signals) at different gate voltages. The source and drain contacts were fabricated by deposition through a shadow mask, the gate contact was realised in the substrate consisting of a p^++^ doped silicon wafer covered with a 100 nm thick gate oxide.

### Biological Tests

Preservatives, antibiotics and antimycotics deposited on round titan plates (Ø 1.0 cm, thickness 0.04 cm) were examined on their antimicrobial efficacy by means of the agar diffusion test. The test organisms, Staphylococcus aureus ATCC 6538 and Candida albicans ATCC 10231, were purchased from the German Collection of Microorganisms and Cell Cultures (Braunschweig, Germany). Muller Hinton Agar Medium, Sodium Chloride Peptone Broth, buffered (SCPB), Tryptic Soy Broth and Tryptic Soy Agar Medium were purchased as dehydrated powders from Merck (Darmstadt, Germany).

The test media were rehydrated with demineralised water and sterilised according to instructions of the supplier. For Candida albicans the Muller Hinton Agar was supplemented with 2% glucose monohydrate prior to sterilisation. After sterilisation and cooling down to approximately 50°C, the agar medium was poured into Petri dishes (Ø 9 cm) in aliquots of 10 ml. Into the coagulating medium titanium plates covered with vacuum deposited antimicrobial substances were placed on their sides in an upright position. Each titanium plate was deposited in the middle of the agar layer of a Petri dish.

Test suspensions of Staphylococcus aureus and of Candida albicans were prepared from overnight cultures at 34°C by floating the cultures using sterile sodium chloride peptone buffer. The cell concentrations in the resulting suspensions were assessed by means of a spectrophotometer and the suspensions were diluted in order to adjust microbial numbers of 5×10^5^ cfu/ml. 0.1 ml-aliquots of the suspensions were used to inoculate 5 ml portions of sterilized and cooled agar medium to achieve test concentrations of about 10^4^ colony forming units per ml. Immediately after inocculation and mixing, these portions of the fluid agar medium were poured into the test dishes in order to get homogenous layers around the titan plates. Then the agar was allowed to gelate.

Control plates were prepared using paper discs (Ø 0.6 cm) which were impregnated with 20 µl of a diluent containing the required test substance. In the case of poor water solubility dimethylsulfoxide was used, hydrophilic substances were dissolved in sterile demineralised water. As final concentration per paper disc, 30 µg of the respective substance were chosen. The discs were placed on the surfaces of inoculated agar medium plates after their gelation. The test Petri dishes were incubated at 35°C for 20 hours and then the extents of the inhibition areas were measured.
